# Process Evaluation of a Randomised Controlled Trial for TeleClinical Care, a Smartphone-App Based Model of Care

**DOI:** 10.3389/fmed.2021.780882

**Published:** 2022-02-08

**Authors:** Praveen Indraratna, Uzzal Biswas, Hueiming Liu, Stephen J. Redmond, Jennifer Yu, Nigel H. Lovell, Sze-Yuan Ooi

**Affiliations:** ^1^Department of Cardiology, Prince of Wales Hospital, Sydney, NSW, Australia; ^2^Prince of Wales Clinical School, University of New South Wales (UNSW), Sydney, NSW, Australia; ^3^Graduate School of Biomedical Engineering, University of New South Wales (UNSW), Sydney, NSW, Australia; ^4^Centre for Health Systems Science, The George Institute for Global Health, Sydney, NSW, Australia; ^5^School of Electrical and Electronic Engineering, University College Dublin, Dublin, Ireland; ^6^Tyree Institute of Health Engineering, University of New South Wales (UNSW), Sydney, NSW, Australia

**Keywords:** process evaluation, digital health, mHealth, acute coronary syndrome, heart failure, smartphone

## Abstract

**Background:**

A novel smartphone app-based model of care (TeleClinical Care – TCC) for patients with acute coronary syndrome (ACS) and heart failure (HF) was evaluated in a two-site, pilot randomised control trial of 164 participants in Sydney, Australia. The program included a telemonitoring system whereby abnormal blood pressure, weight and heart rate readings were monitored by a central clinical team, who subsequently referred clinically significant alerts to the patients' usual general practitioner (GP, also known as primary care physician in the United States), HF nurse or cardiologist. While the primary endpoint, 30-day readmissions, was neutral, intervention arm participants demonstrated improvements in readmission rates over 6 months, cardiac rehabilitation (CR) completion and medication compliance. A process evaluation was designed to identify contextual factors and mechanisms that influenced the results, as well as strategies of improving site and participant recruitment and the delivery of the intervention, for a planned larger effectiveness trial of over 1,000 patients across the state of New South Wales, Australia (TCC-Cardiac).

**Methods:**

Multiple data sources were used in this mixed-methods process evaluation, including interviews with four TCC team members, three GPs and three cardiologists. CR completion rates, HF outreach service (HFOS) referrals and cardiologist follow-up appointments were audited. A patient questionnaire was also analysed for evidence of improved self-care as a hypothesised mechanism of the TCC app. An implementation research logic model was used to synthesise our findings.

**Results:**

Rates of HFOS referral (83 vs. 72%) and cardiologist follow-up (96 vs. 93%) were similarly high in the intervention and control arms, respectively. Team members were largely positive towards their orientation and training, but highlighted several implementation strategies that could be optimised for TCC-Cardiac: streamlining of the enrolment process, improving the reach of the trial by screening patients in non-cardiac wards, and ensuring team members had adequate time to recruit (>15 h per week). GPs and cardiologists viewed the intervention acceptably regarding potential benefit of closely monitoring, and responding to abnormalities for their patients, though there were concerns of the potential additional workload generated by alerts that did not merit clinical intervention. Clear delineation of which clinician (GP or cardiologist) was primarily responsible for alert management was also recommended, as well as a preference to receive regular summary data. Several patients commented on the mechanisms of improved self-management because of TCC, which could have led to the outcome of improved medication compliance.

**Discussion:**

Use of TCC was associated with several benefits, including higher patient engagement and completion rates with CR. The conduct and delivery of TCC-Cardiac will be improved by the findings of this process evaluation to optimise recruitment, and establishing the roles of GPs and cardiologists as part of the model.

## Introduction

Globally, patients with heart failure (HF) and acute coronary syndromes (ACS) are often readmitted into hospital within 30 days (1, 2). Often, this is due to a lack of engagement with outpatient services, which may stem from inadequate coordination, communication or access (3). Readmission rates among Australian patients approach 20% in the first month after discharge for both HF (4) and myocardial infarction (5), although many are preventable (6). For HF alone, readmissions are estimated to carry an annual cost of over $600 million (7). Apart from the natural progression of the disease, contributing factors to readmissions include inadequate treatment of risk factors, such as hypertension, and non-adherence with medications and lifestyle advice. Mobile phone based (mHealth) interventions, which encompasses both short message service (SMS) based, and telemonitoring interventions, have been trialled for patients diagnosed with either ACS or HF, albeit with mixed results. A recent meta-analysis demonstrated that overall, the use of mHealth interventions was associated with an overall reduction in HF hospitalisations (8), but only one study of five showed a statistically significant reduction (9). In ACS patients, the focus of mHealth studies has been medication adherence, with no randomised trials reporting the endpoint of hospitalisation.

A collaboration between the cardiology department at Prince of Wales Hospital (POWH) and the Graduate School of Biomedical Engineering at UNSW Sydney, Australia, resulted in the design of a mobile application (app) named TeleClinical Care (TCC) that aimed to improve patients' self-management, and to provide clinicians with daily home-based readings blood pressure (BP), heart rate (HR) and weight. The data were measured using three Bluetooth-enabled digital devices: a sphygmomanometer, weighing scale and fitness wristband. The data were automatically transmitted to the app and to a web-based server (KIOLA), where a pair of clinicians (a cardiologist and a cardiology nurse practitioner) alternated the role of monitoring readings during business hours. A randomised control pilot trial of TCC was undertaken to compare TCC plus standard care, vs. standard care alone in patients being discharged after a hospitalisation due to either acute coronary syndrome (ACS) or heart failure (HF), and the results are briefly summarised here (*n* = 164, intervention arm *n* = 83, control arm *n* = 81). The average age was 61.5 years in both groups. 78% had a primary diagnosis of ACS, the remainder (22%) having a primary diagnosis of HF. There was no significant difference in the primary outcome (11 readmissions in both groups at 30 days, *P* = 0.97). However, at 6 months, there was a statistically significant difference in total readmissions (41 in the control arm, and 21 in the intervention arm, hazard ratio 0.51, 95% CI 0.31–0.88, *P* = 0.015), as well as readmissions due to cardiac causes (25 vs. 11, *P* = 0.025). There was an improvement in medication compliance as measured by self-reported questionnaire (Morisky-Green-Levine [MGL] score). The proportion of patients who reported good adherence (MGL score 4/4) increased significantly in the intervention arm (48% to 75%, *P* < 0.001). In contrast, this proportion did not significantly change in the control arm (61% to 50%, *P* = 0.19). The overall interaction favoured the intervention arm (P_interaction =_0.002). ACS patients in the intervention arm were more likely to complete CR (20/51, 39 vs. 9/49, 18%; OR 2.9; 95% CI 1.15–7.17; *P* = 0.02). There was no significant difference in other secondary endpoints, including BP, weight, quality of life, patient activation (a measure of patient engagement in healthcare), waist circumference and six-minute walk distance (6MWD), although the loss of data due to cancellation of in-person follow-up appointments during the COVID-19 pandemic reduced the statistical power to detect any differences.

The purpose of providing physiological data to the monitoring team was to identify early deterioration in the patient's condition to manage them safely in the community, thus preventing hospitalisation.

The app also allowed the patient, general practitioner (GP, also known as primary care physician in the United States) or cardiologist to review the readings and also provided educational push notifications for patients. GPs and cardiologists were invited to use the KIOLA server to review their patients. Patients underwent study follow-up at 6 months. The primary endpoint was the incidence of all-cause readmissions at 30 days. Key secondary endpoints included all-cause and cardiac readmissions at 6 months, CR completion and medication adherence.

The typical patient journey involved all patients being encouraged to see their GP within a week of discharge, and their cardiologist within ~30 days. ACS patients would be invited to attend CR, usually commencing 2 weeks after discharge. This program typically involved 12 twice-weekly sessions, although uptake has traditionally been low (10). HF patients are usually referred to the HF outreach service (HFOS). The nurse practitioner or nurse would contact and educate the patient, and perform home visitation if necessary.

Hundred and sixty four patients were recruited during business hours from two metropolitan hospitals in Sydney, Australia, during the pilot study of TCC (81 intervention arm, 83 control arm). Recruitment commenced at POWH in February 2019, and at The Sutherland Hospital (TSH) in August 2019. Recruitment was terminated on March 20, 2020 due to the COVID-19 pandemic, as patients with cardiovascular disease were highly vulnerable to the effects of infection (11), and exposure to research staff was considered an unacceptable risk. TCC was the first cardiac digital health intervention (DHI) trialled at either hospital. Two doctors and two nurses were trained in patient recruitment by observing the recruitment process and reviewing an orientation manual.

In summary, the TCC pilot trial is a complex DHI with a model of care that we hypothesised would improve patient self-management and result in early detection and management of any deterioration for patients with ACS and HF who had been discharged from hospital, thereby, reducing preventable readmissions into hospital at 30 days and beyond. While we found no difference between groups at 30 days, there was a statistical reduction of hospital presentations at 6 months, and improved medication adherence. A mixed-methods process evaluation alongside this pilot randomised trial was conducted with the aim to identify for who, how and why this model of care had an impact on, and in doing so, to identify reasons and mechanisms underlying the variation of outcomes. Additionally, the success of this pilot study has resulted in the planning of a large, fully-powered multicentre RCT, with a planned enrolment of over 1,000 patients (TCC-Cardiac). The process evaluation in this paper aims to identify factors that will optimise the implementation of this larger trial.

## Materials and Methods

The process evaluation was designed in line with guidance published by the Medical Research Council (MRC) for process evaluations (12). Three individuals worked on its development, including two who were involved with the design of the original trial (SO & PI) and one who was not (HL).

Specifically, the aims of the process evaluation were:

1. To identify strategies to maximize patient, team member and site participation in preparation for the large, multi-centre TCC-Cardiac trial.
2. To identify the contextual determinants that influenced the success of the TCC program, specifically
  a) Rates of CR and HFOS referral
  b) Follow-up with cardiologists after discharge
3. To evaluate and explore the engagement of GPs and cardiologists with the TCC model of care and KIOLA server and,
4. To identify the impact of TCC participation on patient self-management.

The inclusion criteria for the RCT included English-speaking patients over the age of 18 who were admitted with ACS or HF and owned a compatible smartphone. Patients from outside Sydney, those who were travelling overseas after discharge, those being discharged to another hospital or an aged care facility and those who could not operate the app or provide informed consent due to language barrier or physical or cognitive limitations were excluded.

Table 1 summarises the data sources and methods used to obtain data for each component of the process evaluation.

**Table 1:** Methods utilised in this process evaluation.

**Aim**	**Sub-aim**	**Method of data collection**
1. To identify methods to maximise patient, team member and site participation in preparation for the large, multi-centre TCC-Cardiac trial	Analysis of screening and recruitment (reach)	A database of patients screened for enrolment was compared against a list of patients and their coded diagnoses provided by the data management team at the respective hospitals, according to the Australian Coding Standards (13).
	An analysis of the training and overall experience of team members	Semi-structured interviews with four team members
	Creation of a checklist to assess any new trial site prior to involvement in TCC-Cardiac	- Lead investigator's own experience - Semi-structured interviews with four team members
2. To identify the contextual factors that influenced the success of the TCC program	CR for ACS patients	CR attendance and completion rates were calculated for each site, for patients enrolled in the trial two months before the cessation of cardiac rehab due to COVID-19 (March 2020).
	HFOS	At recruitment, TCC team members documented if the patient was known to, or referred to, the local HFOS.
	Post-discharge cardiologist consultation	An audit of 20 discharge summaries from each site was conducted to identify the timing of post-discharge cardiologist appointment. The cardiologist offices were contacted to confirm if follow up occurred. If a follow-up range was given, then the longest duration within the range was defined as the prescribed follow-up interval (e.g., “4–6 weeks” would translate as 42 days).
3. To evaluate and explore the engagement of GPs and cardiologists with the TCC model of care and KIOLA server	Identifying attitudes of GPs and cardiologists	- Timestamps from KIOLA records to confirm the number of GPs who accessed the platform. - Semi –structured interviews with three GPs and three cardiologists.
4. To identify the impact of TCC participation of patient self-management and their overall rating of the app	Analysis of quotes from patients regarding a possible improvement of self-care due to TCC	All patients in the intervention arm were asked to complete a questionnaire regarding their experience. Within the questionnaire were the questions “what did you like the most about the TCC app” and “in what ways do you feel like the TCC app benefited you” and responses were reviewed for self-care references. The average overall patient rating out of 5 was calculated.

*ACS, acute coronary syndrome; CR, cardiac rehabilitation; GP, general practitioner; HFOS, heart failure outreach service; TCC, TeleClinical Care*.

### Interviews

Semi-structured interviews were conducted by HL and PI between June and October 2020. We have reported this according to the Consolidated Criteria for reporting of qualitative research (see completed checklist, Appendices A, B) (14). The interview guide (see Appendix C) was designed by consensus discussion between HL and PI. PI closely understood the TCC project, while HL is experienced in qualitative research. All four TCC team members, three GPs and three cardiologists (selected by purposive sampling) were invited by email and interviewed by telephone with verbal consent for audio recording. Face-to-face contact was discouraged due to the COVID-19 pandemic. None of the GPs or cardiologists were involved in the design or day-to-day management of the study.

Domains of inquiry for TCC team members included:

1. The quality of their orientation to TCC.
2. The ‘learning curve' involved in the recruitment process.

Domains of inquiry for GPs and cardiologists included:

1. Whether or not they would use the KIOLA server to access patient data and why/why not?
2. Current and future integration of TCC with their clinical care.

While a series of pre-defined questions was asked, new questions were added to facilitate further discussion of points raised by the interviewee (see Appendix C). There were no refusals to participate. All subjects were assured that their identity would remain confidential. The interviews were transcribed verbatim by PI, and the data were analysed according to the Framework Method for the analysis of qualitative data (15). Specifically, the transcribed text was coded by PI under eight prespecified categories (see Appendix D). Each category was then systematically reviewed. No repeat interviews or transcript clarification were required.

### Analysis

**Figure 1:**
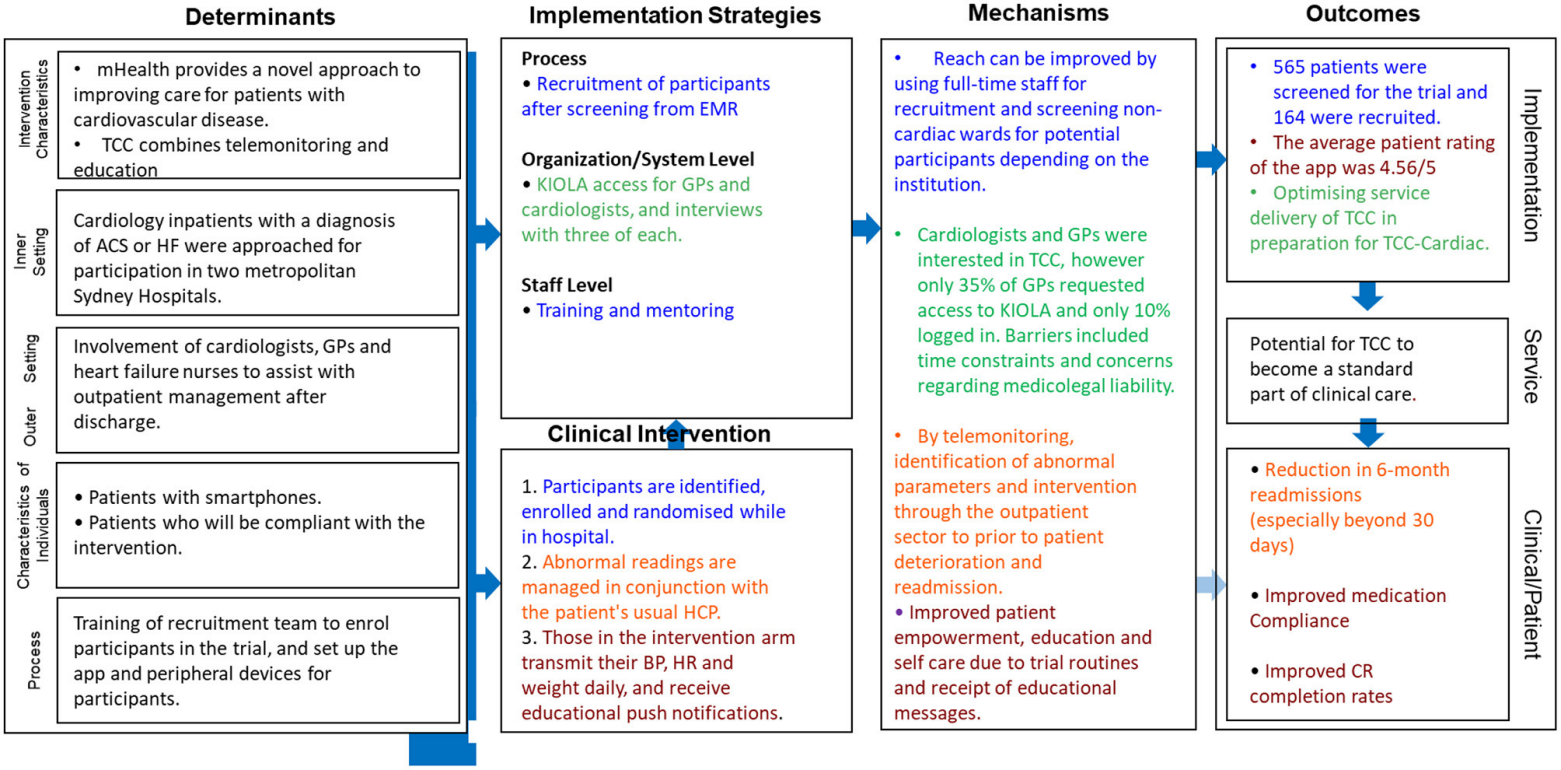
Implementation research logic model (IRLM) for the TCC process evaluation, which describes the contextual determinants, implementation and mechanisms, and outcomes as per the standard process evaluation procedure. The four themes as identified within the refined IRLM relate to the actors involved: the hospital staff providing and delivering the intervention (blue colour within IRLM), the staff monitoring and responding to the patient recordings, healthcare practitioners who care for patients after discharge (GPs, cardiologists and HFOS) and patients participating in the TCC program (brown)”. ACS, acute coronary syndrome; BP, blood pressure; EMR, electronic medical record; GP, general practitioner; HCP, healthcare provider; HF, heart failure; HFOS, heart failure outreach service; HR, heart rate; TCC, TeleClinical Care.

The results of the process evaluation are intended to inform the refined implementation research logic model (16) (IRLM) used to synthesise our findings for the planned TCC-Cardiac trial (Figure 1). The IRLM allows for analysis of a multifocal, complex intervention. It includes the contextual determinants, implementation and mechanisms, and outcomes as per the UK MRC process evaluation guidance, and also embeds the traditional logic model (12, 16). There are four conceptual and theoretical themes contained within the IRLM which relate to the actors involved (patients, TCC recruitment team, TCC monitoring team and health care providers). Each is colour coded, and is linked to the four aims of the process evaluation. Each theme has unique implementation strategies, mechanisms and outcomes. For example, the aim of maximising participation (aim 1) is examined by focusing on the TCC team members responsible for recruitment, represented by blue text in the IRLM.

Aims 2 and 3 focus on GPs and cardiologists (green) and aim 4 focuses on patients (brown).

For statistical analysis, categorical variables were expressed as percentages. Odds ratios were calculated using the Pearson Chi-Square test. Statistical analysis was performed using IBM SPSS Statistics for Windows, Version 26.0 (Armonk, NY: IBM Corp). All analyses applied the intention-to-treat principle.

## Results

### Aim 1: Strategies to Maximise Patient, Team Member and Site Participation in Preparation for the Large, Multi-Centre TCC-Cardiac Trial

#### Reach and Recruitment

Reach is defined as the extent to which the target audience encounters the intervention, in this case, the TCC RCT (17). It is an important concept to identify the transferability of the trial and understanding of the trial outcomes. It addresses the question of “*Did we recruit the types of patients that we intended TCC for*?”

**Figure 2:**
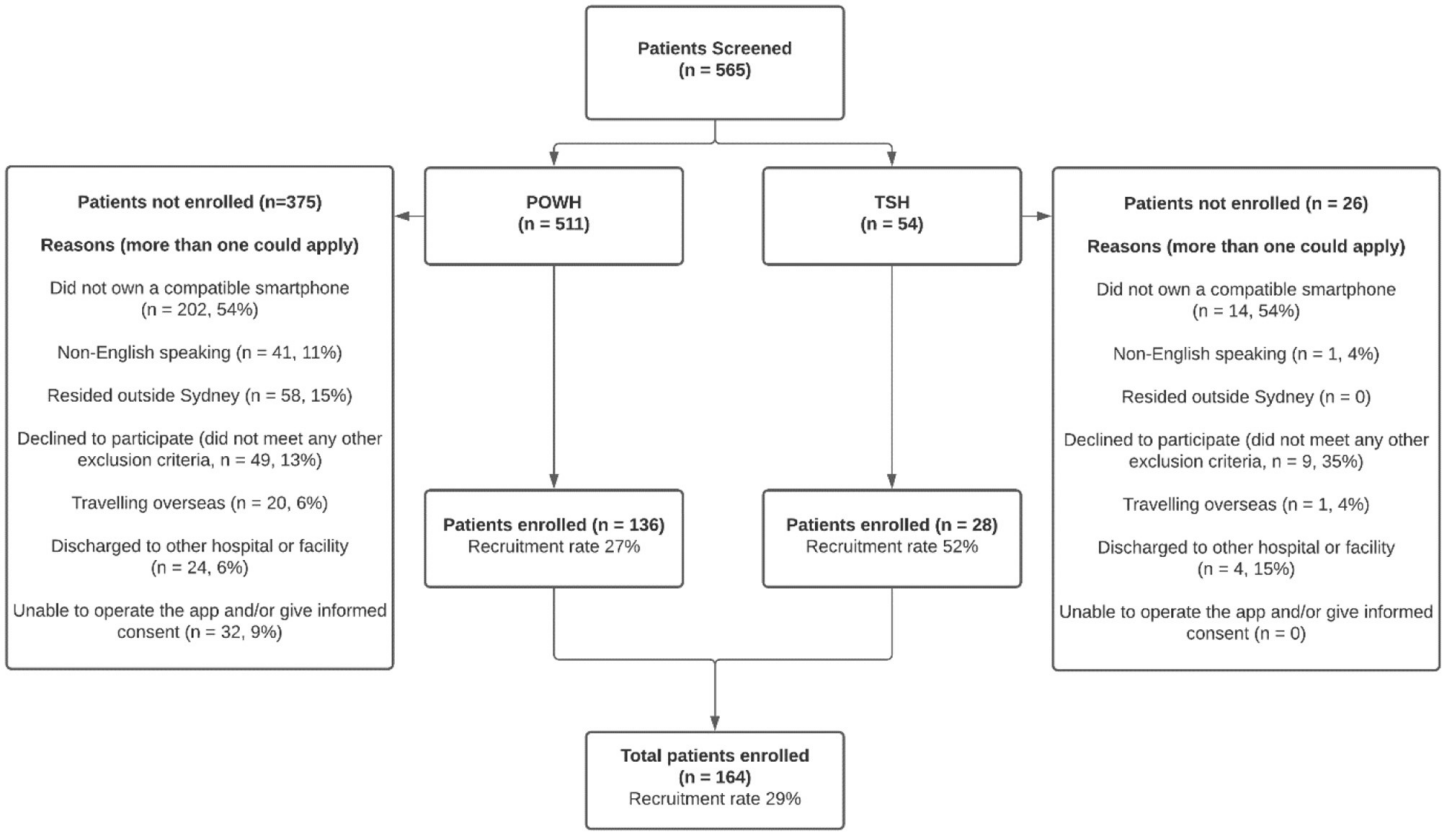
Recruitment patterns between sites. POWH, Prince of Wales Hospital; TSH, The Sutherland Hospital.

A total of 565 patients were screened for eligibility, and 164 (29%) were included in the trial. While this result may appear limited, the major barrier to enrolment was smartphone ownership. Of the 401 screened patients who were not included, mobile phone ownership data was available for 359 (89.5%). Of these, 206 (57%) did not have a smartphone, and 34 (9%) owned an incompatible smartphone. A detailed evaluation of smartphone ownership patterns is beyond the scope of this process evaluation but will occur in a separate analysis. A comparison of exclusion criteria met at each site is presented in Figure 2.

#### Challenges in the Screening and Recruitment Process Affecting the Reach of the Study

During the study period, a search of patients using International Classification of Diseases (ICD) codes revealed 795 patients were admitted to POWH with either an ACS or HF during the study period. Of these, only 394 (49.6%) were screened for eligibility. This was due to discrepancies between the ICD codes and the research team diagnosis. Furthermore, 115 patients were screened for eligibility who were not identified by the hospital dataset, suggesting these patients were also miscoded.

At TSH, 244 patients were admitted with a diagnosis of either ACS or HF according to the hospital dataset during the study period. Twenty five (10%) were screened for eligibility. An additional 29 patients were identified as having an admission diagnosis of ACS or HF, despite not being identified by ICD codes. A total of 54 patients were screened at TSH over seven months. Of these, 28 were recruited, giving an enrolment rate of 52%. The lower proportion of patients screened at TSH was attributable to several factors including:

- The lack of a dedicated full-time TCC staff member at the site, which was particularly challenging when the department was short-staffed.
- Institutional policies that resulted in many patients with cardiac conditions being admitted under alternate specialties such as respiratory medicine and aged care, and thus who were not identified during the screening process (121 of 244 patients, 49.6%).

#### Training and Overall Experience of Team Members

##### Orientation to TCC

All four team members praised their orientation. All benefited from the 1-on-1 approach to observing and then undertaking supervised patient enrolment. The orientation manual was used by 3 of the 4 team members, but only one used it frequently. The others preferred to contact the lead investigator directly for assistance and troubleshooting. Two team members commented that their primary use of the manual was for scoring questionnaire results. One team member commented on the importance of hearing a formal presentation about TCC, as well as being able to use the peripheral devices themselves, to understand the complete picture.

“*The first introduction was when [the principal investigator] gave an orientation talk, which was valuable to understand the greater vision behind TCC and communicated the key messages of the project. Secondly, I was [allowed to use] the actual equipment which was valuable. It was a certainly a sound introduction to the project.” – Team Member (TM) 4*

“*It's a very useful, user friendly manual. I must say I didn't always use it when I could have.” – TM3*

“*To be honest I rarely used [the manual], because it's easier to ask for help!” – TM1*

##### The Learning Curve and Challenges of Patient Enrolment

All team members felt confident in being independently able to enrol a patient after 2–4 attempts. When asked to estimate the time taken to enrol a patient, responses varied (10 min, 30 min, 60–90 min, 120–180 min). Delays in the enrolment process can be divided into trial-related and intervention-related. The major trial-related delay was coordination of the 6MWD test. Intervention related delays included: patients and team-members being unfamiliar with the patient's smartphone, and questions from family members of the patients. App installation and device pairing was considered the most time-consuming part of the enrolment process.

“*[Recruitment] could take a good 2-3 h. I met the patient briefly, explained the study and gave them the information sheet to read. Then I would return, and we would go through the sheet together. During enrolment I had to fill in various parameters, it was not always possible to do all of it at once, especially the 6 min walk test. Sometimes you had to go back and request blood tests that hadn't been done. The enrolment could be done over a number of days. It wasn't really that simple–it could be quite time consuming.” – TM3*

Challenges of completing the enrolment were generally related to time constraints in the busy hospital setting, as patients were often unavailable due to procedures, or were keen to be discharged as soon as possible. Many patients had short inpatient stays.

“*You do have this narrow window between discharge and the patient leaving the hospital. If you're having a busy day, it's hard. I started planning more in advance and trying to predict discharges. I would block out time and discussed it with the patient beforehand to avoid holding up their discharge. With a little trial and error, I managed to smooth it out.” – TM2*

“*Sometimes it was a bit difficult getting all of the data that was needed. We had to wait for procedures and tests to be done, and then had to catch [the patient] before discharge.” – TM1*

Three team members commented on the difficulty in identifying the operating system (OS) of a patient's smartphone, particularly when it was a different OS to their own personal phone.

“*Mostly this was a problem with the Android machines. It was difficult to work out [which] OS it was running and also how to download the app from the Play Store. I just wasn't used to that process” – TM3*

##### Technical Support

While not part of enrolment, the provision of technical support after the patient had commenced the trial was considered challenging by three of the four team members.

“*Troubleshooting technology things over the phone to an elderly person was quite challenging at times- trying to explain step-by-step the things that they needed to do. At times you couldn't get the message across properly and we would do a home visit.” – TM1*

##### Time Commitments

Questions regarding time commitments were targeted at the two team members from TSH who worked on TCC in addition to their full-time clinical duties. They stated the time commitment required was approximately 10–15 h per week, which was challenging. The addition of a second staff member should be considered in this scenario.

“*When you are busy, you cannot enrol the patient in one [session]. You have to repeatedly visit the patient throughout the course of their admission. It is better to do things in cross-section. It would probably take 10–15 h per week to do a proper job of it.” – TM4*

“*There are things that need to be done by a medical professional, and others that can be done by an adjunct staff member. Educating the patient on using the devices and taking the blood pressure correctly all takes a fair bit of time. I do think enrolment needs to be done by a clinical person but spending the time to instal the app and equipment does not.” – TM4*

#### Creation of a Checklist to Assess Any New Trial Site Prior to Involvement in TCC-Cardiac

Prior to recruiting a site for TCC-Cardiac, several key factors must be met (see Appendix D). Broadly, necessary components of the hospital included an inpatient cardiology service with CR and a HFOS. Access to all diagnostic results is necessary, as is sufficient storage and office space. An orientation manual and opportunities to observe the recruitment process and perform it under supervision are considered essential.

### Aim 2: Assessing the Contextual Determinants That Influenced the Success of the TCC Program

#### CR

TCC was designed to work in concert with CR, by reinforcing the educational messages and lifestyle modifications that are recommended by the CR program. Due to the COVID-19 pandemic, however, a full course of CR could only be offered to 100 of the 128 (78%) ACS patients in the trial. Patients in the intervention arm were more likely to complete CR (Table 2).

**Table 2:** Cardiac rehabilitation attendance rate and completion rate.

**Parameter**	**Intervention (*n =* 51)**	**Control (*n =* 49)**	**Statistical analysis**
Attendance rate	28/51 (55%)	21/49 (43%)	NS
Completion rate (attendees only)	20/28 (71%)	9/21 (43%)	OR 3.3 (95% CI 1.01–11) *P =* 0.04
Completion rate	20/51 (39%)	9/49 (18%)	OR 2.9, (95% CI 1.15–7.17) *P =* 0.02

*NS, not significant; OR, odds ratio*.

#### HFOS

The POWH HFOS is managed by a nurse practitioner and a clinical nurse specialist. They conduct home visits and phone calls for over 200 patients. The HFOS at TSH is staffed by more junior nursing staff. They work closely with GPs to instigate management changes. Referral rates are provided in Table 3. There was a high rate of HFOS referral in both trial arms, and no significant difference was found.

**Table 3:** Heart failure outreach service referral rates among patients recruited to TeleClinical Care.

	**HF (all patients)**	**Intervention**	**Control**
Prince of Wales	25/32 (78%)	13/16 (81%)	12/16 (78%)
Sutherland	3/4 (75%)	2 /2 (100%)	1 / 2 (50%)
**Both sites**	**28/36 (78%)**	**15/18 (83%)**	**13/18 (72%)**

*Two patients were referred to other regional services as their residences were out of area*.

*HF, Heart failure*.

#### Post-discharge Consultation With Cardiologists

Forty patients were randomly selected for analysis of follow-up attendance (20 from each site). At the scheduled time of follow-up, one patient was deceased, and two others were hospitalised, leaving 37 patients for analysis. Over 90% of the sampled patients had follow-up with a cardiologist during their time in the trial, with the majority doing so in a timely fashion (Table 4).

**Table 4:** Patterns of cardiologist follow-up recommendations at the time of discharge and attendance.

	**Intervention arm**	**Control arm**	**Total**
*n*	23	14	37
Mean follow up suggestion	42 days	34 days	39 days
Mean actual follow up	40 days	36 days	38 days
Patients who attended follow-up with a cardiologist	22 (96%)	13 (93%)	35 (95%)
Patients who attended follow up 1 week or more after recommended time	7 (30%)	3 (23%)	10 (27%)

### Aim 3: Engagement of GPs and Cardiologists With the TCC Model of Care and KIOLA Server

#### GPs and Access to KIOLA

3/81 intervention arm patients did not nominate a GP. For the other 78 patients, there was a total of 73 GPs, five of whom were uncontactable. Of the 68 contacted GPs, only 24 (35%) requested access to KIOLA, 7 of whom (29%) accessed the server.

#### GP and Cardiologist Perspectives

##### The Low Uptake of KIOLA Among GPs

It was proposed by GP2 that time constraints were the major reason behind the low uptake. Other concerns included issues of medico-legal liability. It was also suggested by GP3 that certain GPs may not be comfortable with new technology.

“*When there are only 15-min slots, it can be quite pressured...the GP would have far less time and be less inclined to look at additional information.” - GP2*

##### The Usefulness of TCC in Clinical Practise

All three cardiologists and two of three GPs felt TCC was likely to be a useful addition to their clinical practise. The main advantage described was having an accurate long-term BP record. GP1 felt that automatic sphygmomanometers were unreliable, and she relied on in-office manual readings. One cardiologist praised the ability to detect asymptomatic atrial fibrillation, which resulted in a significant change in treatment for his patient.

“*It's challenging as the GP to figure out what the patient's blood pressure actually is, based on a single reading” - GP3*

“*Blood pressure is one of the hardest things to get right. A single reading in the office can be meaningless.” - Cardiologist 2 (C2)*

##### Receiving Alerts

*Two* out of the three cardiologists expressed concerns about the volume of alerts that they would potentially receive, but both were still in favour of receiving them.

“*Do I want to be called about every minor abnormality? I think the answer is no. If there is a significant change, and I feel I can have an impact on their therapy, then yes.” – C1*

##### Viewing Patient Data

All three cardiologists and two of three GPs stated they would be interested in viewing a patient's data. Three options were provided to do so – (1) KIOLA access via office desktop computer, (2) viewing the data on the patient's smartphone and (3) receiving a patient summary from the TCC team. The clinicians were asked to select their preferred option.

GP1 was not asked this question based on her previous answers. GP2 stated he would be happy with any option, and GP3 preferred option 1. All three cardiologists preferred a report-based option, generated monthly, in either an electronic or paper form to be stored in the patient's file. This option is to be considered for TCC-Cardiac.

A common theme was the ability to recall which patients were involved in the trial, as patients may not volunteer this during their visit, and thus the data would not be viewed. GP2 stated it should be the patient's responsibility to remind the clinician. C2 suggested that the TCC team contact the cardiologist's rooms so that it is flagged in the patient's file, and C3 suggested a mobile-phone-based reminder of which patients were involved.

##### Which Clinician Should Primarily Manage Alerts?

GP1 was happy that either clinician managing the alerts, with the requirement of correspondence provided to the other. GP2 preferred the cardiologist be responsible, and GP3 felt the GP should be responsible, but would manage the alerts with some guidance from the cardiologist.

Of the three cardiologists, the responses also varied. C1 stated that the GP should be the first point-of-contact, but the cardiologist should be involved if the GP is uncertain how to proceed. C2 felt GPs should ideally be responsible but doubted whether that was practicable. C3 felt it would be situation-dependent.

“*[The responsibility] should go to the cardiologist in heart failure. In ACS, it needs to be a time-based thing. First three months—cardiologist. After that—GP. Early on, [the patient] may not have even seen the GP! They may not be seeing their cardiologist for 9 months.” – C3*

*I think it should go to the GP. Prevention is our job. It would be best if instructions were given by the cardiologist to me, so that I could just follow the plan.” – GP3*

##### Medicolegal Liability

Two of the three GPs were concerned about medicolegal liability. None of the three cardiologists felt this to be a major concern in widespread adoption of TCC.

#### The Impact of TCC Participation on Patients

The app was positively received by patients, with an average rating of 4.56 out of 5. While not directly targeted in the questionnaire, several participants volunteered that TCC impacted their self-care and motivation. A sample of representative answers are provided below:

“*[TCC] was giving me incentive to stay on top of my condition” (male patient, age 61)*

“*It creates a focus on maintaining a healthy lifestyle” (male patient, age 67)*

“*It is something that encouraged me to have a little bit of discipline” (male patient, age 76)*

“*It helped me to feel like I was in control, and was a reminder to look after myself” (female patient, age 57)*

“*It made me accountable for my own readings and checking the progression of my own health” (male patient, age 53).”*

## Discussion

The TCC pilot study demonstrated several significant benefits to participants. There was a statistically significant reduction in total readmissions, driven by a reduction in cardiac readmissions. This finding is of great importance, as although meta-analysis has shown that mHealth interventions are associated with a reduction in HF hospitalisations (8), this is only the second individual RCT to show an impact on hospitalisation. The first was a text messaging intervention implemented in China (9). Other telemonitoring studies in HF have failed to show a benefit in hospitalisation rates (18–20), although one Belgian study demonstrated a mortality benefit (21). In patients with ischemic heart disease or ACS, the impact of telemonitoring on hospitalisation rates has not been examined previously in any mHealth RCT.

This process evaluation aimed to identify the factors required to establish a digital health trial in two metropolitan hospitals that previously had no experience in the field, as well as identifying underlying contextual factors that may have contributed to the results of the trial, and evaluating potential strategies to optimise implementation of the TCC model of care to multiple sites in the planned TCC-Cardiac RCT. The reach of the trial is difficult to quantify due to the lack of a clear denominator in terms of patients admitted with ACS or HF. This is attributed to over-diagnosis, under-diagnosis or miscoding of patients. Patients may have been diagnosed as having an ACS despite not meeting established standard definitions for this diagnosis, and vice versa. This is likely to be a problem in the TCC-Cardiac trial also, and without auditing each patient individually, the true reach of the project cannot be known. When considering screening and recruitment rates at new sites, an understanding of the workforce and institutional admission patterns is required. For example, the tendency of patients with HF to be admitted under specialties other than cardiology at TSH reduced the reach. In order to maximise patient participation, it is recommended that for TCC-Cardiac, that non-cardiac wards are screened for potential enrolments, and that staff can commit 15 h per week for enrolment duties.

Interviews with TCC team members revealed details of the training process, the learning curve, and the challenges of the enrolment process. The answers provided by the team members have been used in the creation of the site setup form. Proposed methods to optimise the orientation experience are summarised in Table 5. Looking ahead to TCC-Cardiac, participating sites must meet several pre-requisites prior to commencement of enrolment. “Usual care” should be standardised. Thus, sites are required to have an inpatient cardiology service, as well as outpatient options for CR, HFOS and cardiologist follow-up. Recruitment for the trial is clearly optimal when there is full-time coverage, as evidenced by the lower recruitment rate at TSH. Clinicians performing recruitment as an addition to other clinical duties may struggle to screen and recruit potential participants, particularly when their other roles are busier than expected, or when they must cover for a colleague on leave. Ideally, 10–15 h per week to recruit participants should be “protected”. Given how patients are often unavailable or inappropriate to approach for recruitment due to their clinical condition, investigations, procedures and discharge planning, this time commitment may be sporadic, rather than continuous, and new team members should be warned of these challenges. It should be noted that one of the major delays during enrolment was the 6-min walk test, which will not be required outside the clinical trial setting.

**Table 5:** Features of the training process for TCC team members.

**Description in the TCC trial**	**Method of optimisation for TCC-cardiac study**
• An orientation manual was used by 3 of 4 team members. It was positively described but not used frequently, except to score questionnaire results and identify smartphone compatibility. • The lead investigator was easily contactable for assistance with recruitment.	• Provide all team members with the orientation manual. • Provide email and phone number of lead investigator so problems can be rectified at short notice. • All team members to receive an orientation lecture, and the TCC app and equipment for self-testing. • Team members to observe two recruitments, and perform two more under supervision.

TCC is designed to support, rather than replace, the benefits of GP care, cardiologist care, CR and HFOS. CR, which comprises education and exercise, is considered a cornerstone of post-infarction care and is recommended for all patients who are admitted with an ACS (22, 23). The CR completion rate prior to COVID-19 at POWH was similar to the previously quoted worldwide average of 20–30% (24). Due to the pandemic, 22% of enrolled ACS patients were denied a full course of CR. Due to randomisation, this is assumed to have affected both groups equally.

Referrals to HFOS were relatively high in both arms (83% intervention vs. 72% control). This was crucial to the success of the trial, as the HFOS was often required to respond to abnormal clinical parameters. TCC is designed to improve the workflow of the HFOS by two specific mechanisms: (i) it allows rapid vital sign assessment of a large population of patients rather than requiring manual phone calls or home visits to gather this data and (ii) identification of stable and unstable patients, which allows for optimal allocation of time and resources to the patients at highest risk for hospitalisation. The combination of these two factors will thus potentially allow an increase in the capacity of the number of patients that can be cared for under the HFOS. Since HFOSs have been shown to improve readmission rates and mortality (25), institutions should be aiming for 100% referral rates, and TCC-Cardiac may potentially provide an incentive to do so. In settings where HFOS referral rates are low, the magnitude of readmission reduction due to TCC will likely diminish.

Follow-up rates with cardiologists were high in a random sample of 37 patients. The typical suggested follow-up time was approximately 6 weeks, consistent with published recommendations (26). Previous work has found that follow-up within 7 days lowers the rates of readmission within 30 days (27), however this is not routine practise in either site, and would have been an important confounder for the primary endpoint of 30 day all-cause readmission.

Given the high rates of HFOS referral and cardiologist follow-up in both groups, it can be concluded that variations in usual care were not responsible for the positive findings in the TCC trial. The mechanism of TCC in improving readmission rates, CR and medication adherence is likely a combination of telemonitoring and improved patient self-care. Several participants expressed that their involvement in the trial created a focus on their condition, lifestyle, and health choices. Several previous mHealth studies have demonstrated an improvement in medication adherence (28–31), typically by motivating patients. Improved adherence is likely a key factor in improving outcomes potentially in many chronic diseases. Improved self-care, medication adherence and CR completion may only develop after several months, which may explain why a reduction in readmissions was seen at 6 months and not at 30 days. Therefore, the primary endpoint of the TCC-Cardiac trial will be all-cause readmissions at 6 months. Whether these benefits persist beyond 6 months remains unknown and requires further study.

### The Role of GPs and Cardiologists

For the TCC-Cardiac trial, it is proposed that the cardiologist be the primary point of contact for the research team in responding to alerts, and that this should be clearly established for each patient. Most GPs who were offered access to KIOLA did not access it. Reasons identified in the series of interviews included time constraints, resistance to change, and concerns over medicolegal liability. Each GP cared for only 1–2 patients in the trial, whereas cardiologists had several more, and over time may become more familiar with the TCC model. A specialist may be less available for urgent and semi-urgent consultations than a GP, however, thus a reasonable alternative would be for the cardiologist to recommend a GP visit if they were unable to see the patient themselves and provide some guidance to the GP in managing the clinical issue. The research team could facilitate this discussion. The best approach, also to be considered for TCC-Cardiac may be to individualise a customizable action plan on a case-by-case basis. This plan could be established by speaking to the patient, the GP and the cardiologist at the point of enrolment. Regardless of which clinician is the primary point-of-contact, correspondence should be provided to the other regarding investigations or treatment changes.

Other considerations for TCC-Cardiac included a robust method of identifying patients who were involved. Ideas proposed included informing the practise secretary to identify the patient prior to consultation, and the provision of a report containing trends in the parameters (weight, HR and BP), delivered every 1–3 months by electronic means. Both are simple modifications, which are to be considered for TCC-Cardiac. Ultimately, since TCC requires the input of the GP and/or cardiologist, it may serve to strengthen the doctor-patient relationship by providing a means of closer monitoring. This, in turn, could lessen the risk of future readmissions. The use of TCC may also improve links between practitioners, if one or both are taking an active role in the management of alerts generated by the program.

### Broader Applications of This Process Evaluation

The COVID-19 pandemic has led to an increase in DHIs, and it is presumed that this will continue after the pandemic's eventual resolution. There is great heterogeneity among DHIs—ranging from simple text messaging programs to more complex telemonitoring solutions such as TCC. Also, the target patient cohort can vary widely, as DHIs can be applied to a large variety of medical conditions, and are inherently scalable to large populations. Therefore, in the digital health sphere, process evaluations are of critical importance and we urge all triallists to consider undertaking them. Several findings of this process evaluation are applicable to the broader digital health context. For example, when assessing the potential reach of mHealth interventions, smartphone ownership may be a rate-limiting factor, and this must be considered. Patients lacking smartphones are at risk of being excluded from beneficial models of care, and strategies are required to address this gap. This may include loaning or rental of smartphones or tablets, with education and instruction in their operation. An understanding of the challenges and time commitments for team-members is also necessary, as for many, this may be their first experience with a DHI.

Further, complex DHIs such as TCC exist within the healthcare ecosystem and involve multiple healthcare practitioners, as well as the patient. In this case, TCC required the input of GPs, cardiologists and HFOS staff. Depending on the nature of the intervention, other medical specialists or allied health staff will be required to interact with patients. Consideration of how the intervention will involve, and be received by, practitioners outside the immediate investigating team is necessary to maximise the benefit to the patient. Investigators should consider whether the intervention will lead to an increased or streamlined workload for these practitioners, and whether care coordination will be improved or complicated, and whether any challenges such as medicolegal liability may be perceived.

DHIs, of any nature, will have an impact on the patient. Potential positive effects include improved medication adherence and self-care, and methods of evaluating these should be considered for all digital health trials. Additionally, when considering the endpoints for digital health trials, these factors should be considered. For example, as medication adherence and self-care improvement take time to develop, clinical outcomes may only prove to be different after several months of using the intervention, rather than immediate. Therefore, endpoints such as 30-day readmissions may not be influenced by certain DHIs, but the trial should not be considered negative if this is the case. Rather, long-term endpoints should be used to adjudicate the efficacy of these interventions, such as 6-month or 1-year readmission rates.

### Limitations

The COVID-19 pandemic resulted in removal of CR from standard care, and resulted in premature termination of enrolment. Thus, the sample size at TSH was smaller than anticipated. Analysis of timing of follow-up was limited to a representative sample of participants rather than all participants. Interviewing a greater number of GPs and cardiologists may have resulted in gathering of further data. Specific questioning of patients regarding mechanisms of benefit such as improved self-care should be considered in the TCC-Cardiac trial. Miscoding of patient diagnoses was also identified which precluded accurate calculation of the reach of the trial.

## Conclusion

The TCC model of care has significant potential for reducing the strain on healthcare systems, as well as empowering patients to achieve better outcomes for secondary prevention of ACS or decompensated HF.

This mixed-methods process evaluation identified differences in the enrolment process and service delivery at both sites involved in the original TCC trial. Thus, it provided a template and pathway for the initiation of new sites into a larger multicentre trial (TCC-Cardiac). This trial is expected to be a pivotal trial into encouraging widespread implementation of this model of care to cardiac patients across the state of New South Wales, provided that clinical benefits are replicated, and that cost-effectiveness is acceptable. Mechanisms of change, such as exploring benefits relating to improved self-care among patients, should be further characterised. This process evaluation also identified options that would streamline the transition of the system from a research project to mainstream clinical practise, and highlights concepts applicable to DHIs outside cardiology.

## Data Availability Statement

The raw data supporting the conclusions of this article will be made available by the authors, without undue reservation.

## Ethics Statement

The studies involving human participants were reviewed and approved by South Eastern Sydney Local Health District Human Research Ethics Committee. The patients/participants provided their written informed consent to participate in this study.

## Author Contributions

PI: conduct of most aspects of data collection and analysis and primary author of the manuscript. UB: data analysis of the patient questionnaire data. HL: interviewed all team members and provided extensive guidance into the concept and design of the process evaluation. SR: supervision of the project from a technical/engineering perspective. JY: assistance with data analysis and supervision of the project from a medical perspective. NL and S-YO: co-supervisor of the overall project. All authors contributed to the conception, design, and performance of the work and have reviewed the manuscript and accept accountability for it.

## Funding

Prince of Wales Hospital (internal department funding)—for conduct of the TCC RCT. Prince of Wales Hospital Foundation—for conduct of the TCC RCT. UNSW Sydney—for conduct of the TCC RCT. NHMRC (scholarship for author PI) (Grant Number: RG181711). National Heart Foundation of Australia (scholarship for author PI) (Award ID: 102356).

## Conflict of Interest

The authors declare that the research was conducted in the absence of any commercial or financial relationships that could be construed as a potential conflict of interest.

## Publisher's Note

All claims expressed in this article are solely those of the authors and do not necessarily represent those of their affiliated organizations, or those of the publisher, the editors and the reviewers. Any product that may be evaluated in this article, or claim that may be made by its manufacturer, is not guaranteed or endorsed by the publisher.
