# Supplementary material for: Process Evaluation of a Randomised Controlled Trial for TeleClinical Care, a Smartphone-App Based Model of Care
Source: Front Med (Lausanne). 2022 Feb 8;8:780882. doi: 10.3389/fmed.2021.780882 (PMC8862755; doi:10.3389/fmed.2021.780882)
Supplement: Supplementary file 1 [file Data_Sheet_1.docx]

9.2 Appendix B – Interviewer data and detailed interview methodology.

Two interviewers participated in this process evaluation. HL is a female researcher with extensive experience in qualitative research. She interviewed the team members, none of whom were known to her. She is a Senior Research Fellow and her qualifications are BA (Hons), MBBS and PhD. PI is a male cardiologist (MBBS, FRACP). He interviewed the GPs and cardiologists. The reasons for all interviews were explained prior to their commencement. No one apart from the researcher and interviewee was present during interviews. Informed consent was obtained from all participants which provided a sample of possible questions. No pilot testing was necessary. No repeat interviews were required. Notes were taken during the interview to assist with follow-up question planning. Interviews ranged in duration from 10 minutes to 60 minutes. Data saturation was not considered likely. No software was required to manage data.

9.2 Appendix C – Interview Guides

**Questions for Team Members**

1. How long were you a part of the TCC trial team?
2. How would you rate your introduction/orientation to TCC? How could this be improved?
3. Rate your confidence levels in:
   1. Describing the trial to patients (i.e. understanding of the trial)
   2. The process of enrolling patients including randomisation (i.e. consent, baseline measurements, randomisation, data entry)
   3. Installing the app, connecting the peripheral devices and testing their functionality?
   4. Identifying and responding to alerts? [where applicable)]
   5. Responding to technical support issues [where applicable]
4. Enrolling patients
   1. How long would it take you to enrol a patient?
   2. How do you think the process could be expedited?
   3. What was the most difficult/challenging part of the enrolment process?
   4. Describe your experience with obtaining baseline measurements
      1. six-minute walk test?
   5. How many hours per day/week did you spend working on TCC?
   6. How many hours per day/week would someone need to dedicate to TCC to be able to completely screen and enrol patients?
5. Learning curve
   1. Would it be possible for someone to learn how to enrol patients independently, or would they need to be shown?
   2. How many patients did you need to observe being enrolled before you were confident to do it unsupervised?
   3. Do you feel you could act as an “independent” researcher for the TCC trial? How long did it take for you to feel this way?
   4. Do you think you would be able to teach a new person to do the same job as you did for TCC?
6. The “Operations Manual”
   1. Rate the manual in terms of how useful it as
   2. Was the manual clear, easy-to-understand and comprehensive?
   3. Is there anything missing from the manual/how could it be improved?

**Questions for GPs and Cardiologists**

1. Would you want access to KIOLA?
2. Is there a time/log-in burden
3. Why do you think the majority of GPs did not use KIOLA? (GPs only)
4. Whose responsibility is it to manage alerts?
5. How would you like to view the data – on your computer, on the patient’s phone or on paper?
6. How can we better integrate TCC into standard care? (Cardiologists only)

**9.3 Appendix D– Coding Tree for Interviews**

Interviews were performed with TCC team members, GPs and cardiologists. The responses were categorised under the following headings, and an explanation of why each was chosen is provided.

**9.3.1 Recruitment of participants**

9.3.1.1 Introduction and orientation

Team members introduction to their role with TCC was assessed, to help identify whether this introductory process could be improved for the TCC-Cardiac trial.

9.3.1.2 Recruitment
The process of participant recruitment was analysed from the perspective of team members to identify how comfortable they were with performing recruitment, as well as identifying methods of streamlining the process for the TCC-Cardiac trial.

9.3.1.3 Operations Manual

The operations manual required evaluation by the TCC team members, as a similar manual will be prepared for the TCC-Cardiac trial, which likely will have many more individuals requiring to refer to it.

9.3.1.4 App Setup and Peripherals

The process of app and peripheral setup was felt to be the most difficult aspect of enrolment, and we wished to examine how difficult this was for team members.

**9.3.2 During participation**

9.3.2.1 Technical support

TCC team members acted as the first-line of technical support, and they did not have a technical background. Therefore, the aim was to understand whether medical and nursing personnel could provide this service, and what their limitations were.

9.3.2.2 Back-end monitoring

The purpose of this was to learn about the requirements and intricacies of performing back-end monitoring.

**9.3.3 – Questions for Clinicians**9.3.3.1 Access to KIOLA and results

We feel that for the TCC model to optimally function, there needs to be engagement of the patients’ usual healthcare providers with the system. Thus, we enquired about whether clinicians would use the system. Responses regarding receiving summary data in report format were also categorised here.

9.3.3.2 Barriers

Potential barriers to more frequent engagement with the system were examined, to identify how best to optimise TCC in the TCC-Cardiac trial.

**9.3.4 – Other**

All responses that were not coded under the above eight categories were listed here.
