# Supplementary material for: Process Evaluation of a Randomised Controlled Trial for TeleClinical Care, a Smartphone-App Based Model of Care
Source: Front Med (Lausanne). 2022 Feb 8;8:780882. doi: 10.3389/fmed.2021.780882 (PMC8862755; doi:10.3389/fmed.2021.780882)
Supplement: Supplementary file 2 [file Data_Sheet_2.pdf]

TeleClinical Care

Site Setup Form

Site Name:____

All aspects need to be confirmed as 'Yes' prior to recruitment commencing at the site.

Site Pre-requisites: The site must meet all of the following criteria.

**Components**	**Yes**	**No**
The hospital provides an inpatient cardiology service		
A heart failure outreach program is affiliated to the site		
Cardiac rehabilitation is offered by the site		
Ethics approval has been secured for the site		
Governance approval has been secured for the site		

Site Educational Components: All of the following groups should have been introduced to TCC either by way of a presentation at a departmental meeting, in-service or a dedication discussion.

**Components**	**Yes**	**No**
TCC site team members		
Hospital cardiologists		
CCU and cardiology ward (including the nurse unit managers of CCU and cardiology ward)		
Cardiology junior medial staff (ATs, BPTs and JMOs)		
Cardiac rehabilitation staff		
Heart failure outreach staff		

TCC Team Member: Each site must have at least one individual who will be committed with the task of enrolling and 'onboarding' patients to the TCC system.

**Components**	**Yes**	**No**
Available at least for 15 hours per week		
Has a medical or nursing background		
Access provided for REDCap		
Access provided for KIOLA		
Access provided for EMR and ability to add-on blood tests		
Access provided for echocardiography results		
Access provided for angiography (cath lab) results		

TeleClinical Care

Site Setup Form

Site Name:_______________________________________

Training and Assistance for TCC Team Member

**Components**	**Yes**	**No**
Team member has received the peripherals		
Team member has observed the enrolment of 2 patients		
Team member has recruited two patients under supervision		
Team member has a copy of the *Operations Manual*		
Team member is confident in assessing phone compatibility		
Team member is confident in connecting peripheral devices		
Team member has been given contact details for technical support		
Team member has been given contact details for research support		

Office Space: A lockable office space is required for TCC.

**Components**	**Yes**	**No**
A lockable office is available		
The office has enough room for storage of patient files and peripheral devices		
Computer for TCC use		
Printer for TCC use		
An iPad or mobile phone is provided for formatting of Mi Bands		

Equipment: The following items are required for enrolment

**Components**	**Yes**	**No**
Sufficient scales, BP machines and Mi Bands to meet recruitment target		
A wall-mounted stadiometer to measure height		
A 'master' set of scales and BP machine		
A measuring tape for waist circumference		
A six-minute walk test track with markers		

Assessor Name: ____________________________________

Assessor Signature: ____________________________________
